# Dental caries: relevance of a correct clinical diagnosis

**DOI:** 10.21142/2523-2754-1202-2024-192

**Published:** 2024-06-27

**Authors:** Martin Andrés Chávez-Méndez

**Affiliations:** 1 Universidad Científica del Sur. Lima, Perú. mchavezme@cientifica.edu.pe Universidad Científica del Sur Universidad Científica del Sur Lima Peru mchavezme@cientifica.edu.pe; 2 Universidad de Salamanca. Salamanca, España. Universidad de Salamanca Universidad de Salamanca Salamanca Spain

According to the WHO, three-quarters of the population suffers from tooth decay, more than 3.5 billion people worldwide.^1^ Although dental caries is a well-known disease, proper diagnosis has some grey areas that affect treatment and care, making proper rehabilitation of dental caries difficult for patients.

Conventional methods for diagnosing dental caries, such as tactile examination and visual inspection, continue to be widely used in clinical practice. However, the sensitivity and specificity of this method are questionable and will greatly depend on the working conditions in which the professional finds himself. The conventional evaluation is usually complemented with radiographic examinations (periapical and bite-wing x-rays) that will help confirm advanced dental caries lesions or with a view to pulp involvement of the tooth. The latter is a more sensitive but less specific method (especially when there are initial carious lesions), although together with the tactile examination and visual inspection, it considerably increases the success of the clinical diagnosis. ^(^^2^

Currently, contemporary practical approaches to addressing this disease, such as the non-invasive, microinvasive, and minimally invasive, are based on risk assessment and early detection and prevention of dental caries. ^3^ Caries risk assessment is related to the restorative effect and prognosis of the affected tooth, jointly encompassing the prevention of the disease, developing a treatment plan, and reassessing the risk after treatment. There are several dental caries risk assessment systems around the world, including the American Dental Association (ADA) Caries Risk Assessment, Caries Risk Assessment Tool (CAT), Caries Management by Assessment Risks (CAMBRA), and the Cardiogram.^4^ On the other hand, importance should be given to the systematization of the clinical diagnosis of dental caries. For example, the criteria established by the International Caries Detection and Assessment System (ICDAS) help in the detection of carious lesions from D1 (first visible change in enamel) to D6 (extensive cavitation within visible dentin). Even when used by inexperienced examiners, ICDAS broadens the spectrum of dental caries diagnosis in clinical, research, teaching, and epidemiological settings.^5^

The selection, criteria, or diagnostic system must also be a balancing act in clinical decision-making. Each diagnostic criterion/dental caries index is good for its intended purpose and requires ongoing training by the oral health professional. If we want to change the negative rates of the most prevalent oral disease, we must begin to take our diagnostic consultation with the relevance it deserves. Adequate training will increase the degree of assertiveness we have when deciding which phase of the disease is progressing, and consequently, act better while being considerate of the tooth to be treated.
